# Coronaviruses: Is Sialic Acid a Gate to the Eye of Cytokine Storm? From the Entry to the Effects

**DOI:** 10.3390/cells9091963

**Published:** 2020-08-25

**Authors:** Przemyslaw Wielgat, Karol Rogowski, Katarzyna Godlewska, Halina Car

**Affiliations:** 1 Department of Clinical Pharmacology, Medical University of Bialystok, Waszyngtona 15A, 15274 Bialystok, Poland; hcar@umb.edu.pl; 2 Department of Experimental Pharmacology, Medical University of Bialystok, Szpitalna 37, 15295 Bialystok, Poland; karolrogovsky@gmail.com; 3 Department of Haematology, Medical University of Bialystok, M. Sklodowskiej-Curie 24A, 15276 Bialystok, Poland; kasia89@piasta.pl

**Keywords:** coronavirus, MERS-CoV, SARS-CoV, SARS-CoV-2, sialic acid, Siglec

## Abstract

Coronaviruses (CoVs) are a diverse family of the enveloped human and animal viruses reported as causative agents for respiratory and intestinal infections. The high pathogenic potential of human CoVs, including SARS-CoV, MERS-CoV and SARS-CoV-2, is closely related to the invasion mechanisms underlying the attachment and entry of viral particles to the host cells. There is increasing evidence that sialylated compounds of cellular glycocalyx can serve as an important factor in the mechanism of CoVs infection. Additionally, the sialic acid-mediated cross-reactivity with the host immune lectins is known to exert the immune response of different intensity in selected pathological stages. Here, we focus on the last findings in the field of glycobiology in the context of the role of sialic acid in tissue tropism, viral entry kinetics and immune regulation in the CoVs infections.

## 1. Introduction

According to the International Committee of Taxonomy of Viruses, the subfamily of *Coronavirinae* comprises four genera of coronaviruses (CoV), characterized by high biodiversity and infectious potential [1]. The epidemiological observation in the last decades focused on the invasion of CoVs revealed that α-, β-, γ- and δ-CoVs, cause in mammals and birds a respiratory and intestinal infections that are mainly mild and asymptomatic, but severe and fatal courses were also observed [2]. In the last years, the extensive studies on the highly pathogenic human coronaviruses, especially on SARS- and MERS-CoVs helped to understand their biology but has also driven the discovery of new therapeutic strategies in case of epidemics [3,4]. However, a novel CoV named SARS-CoV-2 was described as the causative agent of the severe acute respiratory syndrome with high invasiveness and mortality, and disease it causes, called COVID-19, was officially declared a pandemic by the World Health Organization (WHO) [5,6,7]. In the face of the massive SARS-CoV-2 invasion, the state of knowledge of CoVs turned out to be insufficient in the field of their structure, infection mechanisms, serology, epidemiology and, finally, effective therapy.

The CoVs belong to the enveloped viruses with single-stranded large RNA genome sizing about 30,000 nucleotides [8]. The viral particle contains several classes of proteins that play structural and non-structural functions. The non-structural proteins belong to the family of replicases that are processed by proteases into 16 proteins functionally involved in genome transcription and replication [9]. Among four structural proteins, nucleocapsid (N) protein form complexes with RNA, whereas spike (S), membrane (M) and envelope (E) proteins participate in viral envelope formation, mediates attachment to the host cell surface receptors and may suppress the host immune response [10,11]. Given the invasive potential, the S protein play pivotal role in the fusion between the viral and host cell membranes, and in some CoVs interferes with interaction between infected and surrounding uninfected cells. The mechanisms underlying the attachment of virus via S protein recruit molecular systems which are specific feature of each CoV genera and a potential candidates in targeting therapy that may prevent a virus from being able to enter a cell. The N-terminal domain (NTD) and C-domain, called receptor binding domain (RBD), of S protein recognize and bind host membrane proteins [12]. As shown previously, the infection with morbid and deadly human CoVs, MERS- and SARS-CoV, is mediated by the high-affinity interaction between viral RBD and respectively, dipeptidyl peptidase 4 (DPP4) and angiotensin-converting enzyme 2 (ACE2) in host cells [13]. However, there is evidence that the main viral entry mechanisms may be supported by sialic acid-based machinery resulting in high diffusion speed [14,15,16]. Given that the sialoglycans are a widespread component of cellular glycocalyx in various tissues, their possible regulatory function in CoVs can open new view on predictive diagnostics and future therapy. It is particularly important in the context of SARS-CoV-2 infections characterized by an overzealous and unexpected immune response contributing to severe acute respiratory syndrome and concomitant highly limited and not fully established drug therapy. This paper briefly focuses on the engagement of sialic acids in tropism and infectivity of CoVs, and their role in the regulation of overreaction of the body’s immune system.

## 2. Sialic Acids in CoVs Infections

### 2.1. Sialic Acids–Structural and Functional Players in the Epidemiological Events

Sialic acids are the nine carbon keto-sugars covalently attached to the terminal end of glycoconjugates on the cell surface and secreted glycoproteins and glycolipids. The biological and physical properties of sialoglycans, including the negative charge and high hydrophilicity of cell membranes, regulate cell-cell and cell-extracellular matrix interactions [17,18,19,20]. Due to sialic acid glycotopes ubiquity it is biologically important in biological recognition and immune responses [21,22,23,24,25,26,27,28]. According to Läubli and Varki sialoglycans expressed in the mammalian glycocalyx can be considered as self-associated molecular patterns (SAMPs) that can interact with the individual immune receptors presented on the same cell membranes and orchestrate inflammatory reactions within damaged tissues [29]. In contrast to SAMPs, the pathogen-associated molecular patterns (PAMPs) express the ability to recognize several immune receptors that underlie the mechanisms of chronic inflammation and neurodegeneration, as well as impaired immune surveillance in pathogen infections. The recruitment of the interplay between sialic acids and sialic acid-binding immunoglobulin-like lectins (Siglecs) underlie the proinflammatory signaling in response to some pathogens eg. *Haemophilus influenzae* and *Escherichia coli* [30,31,32]. In the field of virology, sialic acids were the first identified virus receptors that facilitate post-attachment events in the entry process [14]. The invasion by recognizing and binding to cell surface sialic acids, and in consequence fusion with the cellular membrane, is one of the best-established mechanisms of viral entry in host cells, as demonstrated with highly pathogenic influenza A, -B, and -C strains in vivo and in vitro studies [33,34,35]. Depending on the serotype of envelope glycoproteins that interact selectively with sialoglycans of the host, viruses can recognize and bind α2-3-, α2-6- and/or α2-8-linked sialic acid moieties, however, these preferences are additionally limited by natural host origin. For example, the receptor-binding specificity of human influenza viruses is focused preferably on α2-3- and α2-6-linked sialic acids which predominates on epithelial cells in the nasal mucosa, pharynx, larynx, trachea and ocular cells and strongly correlate with clinical symptoms [36,37,38,39]. The binding of α2-3- and/or α2-6-linked sialic acids as cellular receptors recruited in the host cell entry processes was described in several viral families isolated from humans, including *Adenoviridae*, *Picornaviridae*, *Reoviridae*, *Paramyxoviridae* and *Polyomaviridae* [40,41,42,43,44]. In the context of the virus-host interaction, some viruses genera employ the sialic acid- and Siglec-based mechanisms that avoid or deactivates host defence after pathogen invasion and underlie in the pathogenesis of acquired immune deficiency syndrome (AIDS) [45,46,47,48].

### 2.2. Coronavirinae-Obviousnesses and Surprises

Individual coronaviruses use specific cell receptors, which was used in differentiation and taxonomic classification to one of four genera. The ability to recognize host cell receptors by specific types of viruses is not synonymous with determining their pathogenic properties. It has been shown that NL63-CoV, one of seven known CoVs to infect humans, exerts lower affinity for human ACE2 (hACE2) than SARS-CoVs. These differences are related to the primary and secondary structure of S protein and determinate the clinical course of infection [49]. The infection of the upper respiratory tract with the low pathogenic CoVs including 229E-CoV, OC43-CoV, NL63-CoV, and HKU-CoV is manifested by the mild and cold-like symptoms. In contrast, highly pathogenic MERS-CoV, SARS-CoV and SARS-CoV-2 cause atypical pneumonia with the high rate of mortality [2]. The pathological changes in lungs following SARS- and MERS-CoV infection are accompanied by an excessive immune response and described as “cytokine storm” [50,51,52]. The clinical and experimental observation showed that individuals with severe or lethal SARS present very low serum level of anti-inflammatory IL-10 but high levels of proinflammatory cytokines and chemokines, e.g., IL-1, IL-6, IL-12, CCL2, CXCL10 and CXCL9, in comparison to patients with mild disease [53]. These changes were correlated with increased populations of neutrophils and monocytes in peripheral blood and lungs of infected individuals. In line, the in vitro studies with cultured monocytic THP-1 cell line and human peripheral blood monocytes infected with MERS-CoV confirmed the up-regulated production of pro-inflammatory factors. In animal models employed to study SARS, young mice developed lethal disease featured by pulmonary oedema, diffuse alveolar damage and excessive influx of monocytes/macrophages accompanied by delayed interferone-α/β (IFN-α/β) response, whereas targeting IFN signaling showed protective effects and better prognositic outcomes [4].

One of the greatest difficulties in the management of SARS-Cov-2 infection that underlie COVID-19 pandemic is the not fully understood hypersensitivity or relative resistance in some individuals in the context of the immune response [54]. These differences are genetically dependent and closely related to the structure and function of particular immune receptors. It has been shown that the polymorphisms of Toll-like receptor 1 (TLR1) and TLR4, linked to single nucleotide polymorphism, predispose individuals to “cytokine storm” in response to bacterial products [55,56]. The level of cytokine and chemokine expression results from the interplay between PAMPs and the pathogen recognition receptors (PRRs) of the host. The PAMPs-PRRs axis is featured by high specificity of recognition and interaction capacity between a pathogenic molecule (microbial, protozoan or viral) and engaging immune receptor. In result, the enhanced immune signalling pathways lead to a stage of hyperresponsiveness to PRRs agonists [57]. It has been shown that respiratory syncytial virus (RSV)-infected airway epithelial cells become hypersensitive to endotoxin by enhancing TLR4 expression with production of IL-8 and tumor necrosis factor α (TNFα) as well as increased mitogen-activated protein (MAP) kinase activity [58].

As suggested above, sialic acid-based pattern recognition by Siglec receptors can be considered the axis of SAMPs/PAMPs/PRRs in self-nonself discrimination by the innate immune system. The sialic acid-Siglec immune checkpoint has been described as part of the molecular mimicry system in various bacterial, viral and malignant invasions, but their involvement in CoVs infection and related pathologies is not fully understood [28]. However, extensive research on the COVID-19 pandemic has shed new light on CoVs biology, in particular on infection mechanisms and their importance in prognosis and therapy.

In the following we focus on the involvement of sialoglycans in the human CoVs biology, including the highly invasive species of the genus Betacoronavirus, in the field of mechanisms of host cell infection and the control of the immune response.

**Figure 1:**
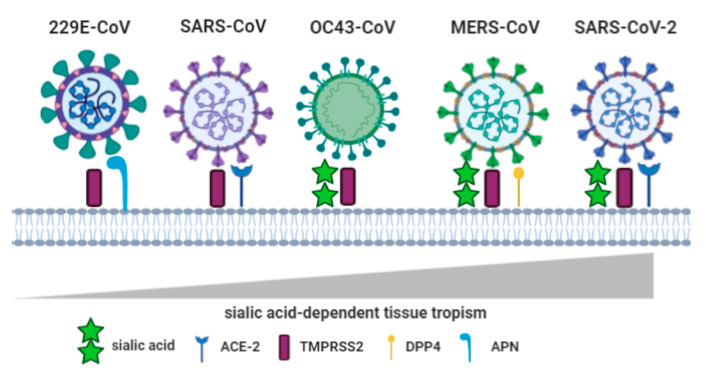
Receptor recognition pattern during the CoVs infections. The CoVs invade host cells through the attachment, binding and entry mechanism based on the sialic acid and protein receptors. After sialic acid-mediated virus attachment and its spike protein activation by transmembrane serine protease 2 (TMPRSS2), the entry event is associated with the binding of specific protein receptor: ACE-2 (angiotensin converting enzyme type 2); DPP4 (dipeptidyl peptidase 4); APN (aminopeptidase N). The ability to recognize sialoglycans determines virus tissue tropism and clinical manifestations among infected organs.

The sialic acid binding capacity is one of many determinants of biological diversity in the family of CoVs [14]. Several types of CoVs infect the host cells in a sialic acid dependent manner, however the mechanisms of virus entry are different than those described in influenza viruses A and B. In contrast to influenza A and B viral HA and NA, the corresponding mechanisms presented by CoVs are based on enzymatic activities of hemagglutinin esterase (HE) and hemagglutinin esterase fusion proteins (HEF) that differ from each other with the ligand bound in the opposite orientation [59]. Among CoVs, the lineage A of the beta-CoVs, such as human OC43-CoV and HKU1-CoV, use 9-*O*-acetylated sialic acid (9-*O*-Ac-sialic acid) as a substrate for HE. The detachment of O-acetyl residues promotes the release of viruses from the host infected cell and prevents the formation of virus aggregates [60]. Interestingly, the transmissible gastroenteritis virus (TGEV) enter their host cells via double mechanism based on binding of sialic acid and aminopeptidase N (APN), whereas its respiratory variant, the porcine respiratory coronavirus (PR-CoV) lacks HA activity and sialic acid binding capacity [61,62]. Moreover, several members of CoVs genera, such as human respiratory 229E-CoV and NL62-CoV, enter their host cells in sialic acid independent manner and recruit only the APN [63] (Figure 1).

### 2.3. MERS-CoV versus SARS-CoV—One Genus and Two Scenarios

Despite some similarities confirmed by genomic and phylogenetic analyses, MERS- and SARS-CoV differ from each other in several structural features that are crucial for infective potential [64]. As highly pathogenic human β-CoVs, they cause a wide spectrum of clinical manifestations in respiratory tracts, however, the rate of clinical changes seem to be unfavorable in MERS-CoV infections [4]. While the clinical course of SARS resulted in acute respiratory illness presents in three distinct phases, the MERS clinical manifestations in respiratory tracts rapidly progress to pneumonia with a 35% overall mortality rate [65]. Comparative studies from human autopsies and animal models of SARS and MERS strongly suggest a pathogenic role for “cytokine storm” derived from inflammatory monocyte-macrophages (IMMs) and neutrophils. In line with this, enhanced serum cytokine and chemokine levels during MERS-CoV infection strongly correlate with increased neutrophil and monocyte numbers in the lungs and the peripheral blood, indicating a possible role for these cells in described pathology [4,66].

As mentioned above, the attachment and fusion with the cell membrane of the host is critical step of viral infection. In the consequence of the encounter of the target cell, the S protein is divided between S1 and S2 subunits responsible for receptor binding and membrane fusion, respectively. In the most CoVs, the C-terminal domain of S1 subunit recognizes specific host receptors, whereas N-terminal domain, depending on the virus type, recognizes specific sugar molecules, and thereby facilitate the initial binding of viral particles to the cell surface. In SARS-CoV, the S protein is cleaved into S1 and S2 subunits by host transmembrane protease serine 2 (TMPRSS2) [67,68,69]. To enter host cells, virus utilizes human ACE2 as the functional receptor recognized and linked via viral RBD of S1 subunit. This mechanism was confirmed in multiple in vitro studies with naturally ACE-2-expressing colonic epithelial LoVo cells and alveolar epithelial A549 cells [70,71]. Whereas the structure of RBD is systematically studied, the NTD of SARS-CoV is not fully characterized. However, the current state of structural analyses suggests that SARS-Cov, unlike other β-Covs, does not recognize glycans, including sialic acids, via NTD in S1 subunit [72]. For MERS-CoV, human lung epithelial cells susceptibility correlates with DPP4 expression in these cells. Analysis of MERS-CoV S1 subunit structure by cryoelectron microscopy showed four individually folded domains (S1A-S1D) implicated in receptor binding. The engagement of binding domain closely depends on virus species [73]. Among β-CoVs, low invasive bovine CoV (BCoV) and human OC43-CoV recruit S1A domain for binding of sialic acid-based entry receptors [74]. Binding of MERS-CoV to the cell surface entry receptor DPP4 occurs via S1B whereas sialic acid-binding activity was assigned to S subdomain S1A. In addition, the analysis with 60-metric lumazine synthase nanoparticle presenting S1A domains revealed high preference for α2.3-linked over α2.6-linked sialic acids. The importance of sialic acid in the mechanism of MERS-CoV infection was presented by Li et al. [73]. They showed that lower sialylated Vero cells exert lower susceptibility to MERS-CoV infection compared to highly sialic acid expressing human airway Calu-3 epithelial cells. Moreover, enzymatic depletion of sialic acids by neuraminidase from the cell surface or their modification by 9-*O*-acetylation or 5-*N*-glycolylation hindered or dampened these interactions and thereby viral entry [73]. As demonstrated in influenza viruses infections, the sialic acid-binding preference and distribution of sialoglycans is a critical regulator of viral tropism. Therefore, site of MERS-CoV replication colocalize with α2,3-linked sialic acid in vivo. According to Li et al., the differential distribution of α2,3-linked sialic acid in humans and camels correlates with predominant sites of MERS-CoV replication in the lower and upper respiratory tracts, respectively [73]. Finally, the identified MERS-CoV interaction with the host sialosides in the dual-receptors binding mechanism, has been characterized by fast kinetics and was required for viral entry [75].

### 2.4. SARS-CoV-2 versus SARS-CoV—Are Sialic Acids Really Important?

The comparing analysis of genetic sequences revealed that SARS-CoV-2 shared about 79% of its genome with SARS-CoV [76,77]. In addition, the overall sequence similarity for the whole S protein was 78% and 76% for RBD [78]. The close identity between S1 subdomains indicate that SARS-Cov-2 also utilizes ACE2 as the receptor. Despite similarities, SARS-CoV-2 show several structural differences that may influence infection kinetics and clinical symptoms in individuals. First, quantification of the kinetics of ligand-receptor interaction by surface plasmon resonance showed that SARS-CoV-2 binding to ACE2 is about 20-fold higher compared to SARS-CoV [79]. Second, clinical course of COVID-19 in some patients range from mild symptoms to deadly “cytokine storm”, however, asymptomatic individuals, similar to those in MERS, may pose a significant public health problem [80]. To better understand the mechanisms underlying these phenomenon, the total genomic sequence of human CoVs was compared with the library of viruses. Studies on the complementarity of SARS-CoV-2 S protein identified another possible binding region responsible for the interaction with the host receptors. Despite the SARS-CoV-2 is less genetically similar to MERS-CoV (about 50%) than SARS-CoV (about 79%), it has been hypothesized that infection with both SARS-CoV-2 and MERS-CoV depends on similar mechanism of the attachment to the host cells that facilitates virus entry and mediates the immune response [81,82,83]. The structural properties of several SARS-CoV-2 and MERS-CoV strains were analysed using CROSS*align*, a tool for prediction of the structural similarities of two RNA profiles of different length. In the preliminary studies, group of Vandelli have found that the structural region between nucleotides 23,000 and 24,000 of S region that corresponds to amino acids 330–500 of S protein domain, is highly conserved across SARS-CoV-2 strains and responsible for ACE2 binding. However, the structural profile in SARS-CoV-2 at nucleotides 22,500–23,000 corresponds to amino acids 243–302 that share identity in MERS-CoV, but not in SARS-CoV, and bind to sialic acids during virus attachment to the host cells [82]. This observation was confirmed by Milanetti and coworkers in the preliminary analysis using quantitative assessment of the geometrical shape complementarity between interacting proteins. They used experimental structures of SARS-CoV-2, MERS-CoV and SARS-CoV S protein and sialic acid complexes, extracted their binding regions and described through Zernicke descriptors. The selected region in the analyzed viruses expressed the highest similarity when SARS-CoV-2 and MERS-CoV were compared. In addition, the MERS-CoV S protein region interacting with sialic acids and its analogue in SARS-CoV-2 showed electrostatic similarity whereas the compared region in SARS-CoV present not compatible charge that disturb the interaction with sialylated receptors. Milanetti suggest that the sialic acid binding capacity in SARS-CoV-2 seem to be a part of complex cell entry mechanism that explain highly effective infection of the upper and its progession in low airways [83]. This hypothesis seem to be confirmed by the findings of Fantini and coworkers [81]. They identified the specific area of NTD that interacts with oligosaccharide part of ganglioside GM1. Molecular and structural studies revealed that amnio acid residues 100–175 of the NTD, especially Phe-135, Asn-137 and Arg-158 are critical for the interaction with the host GM1 molecule.

Beside the upper respiratory tract, the eye exposure to infectious fluids may be associated with increased risk for SARS-CoV-2 transmission. According to goup of Craeger the conjunctival and corneal epithelia and the nasolacrimal system contain both α2-3-linked and α2-6-linked sialic acid receptors preferentially interacting with numerous human viruses [84]. The eye contamination with CoVs was confirmed by Loon and coworkers using PCR analysis of tears samples collected from probably positive patients [85]. Recently, the clinical observation by Colavita et al. have revealed that SARS-CoV-2 RNA was detected in ocular swab whereas it was undetectable in nasal swab form patient diagnosed for COVID-19 [86].

As mentioned above, changes in sialylation pattern are of particular importance in glycan-dependent biological recognition and contribute to immune disturbances and related pathologies. According to WHO reports, severe symptoms and worse prognosis among patients with COVID-19 are statistically more frequent among individuals with cardiovascular and chronic respiratory diseases than those with no pre-existing chronic medical disfunctions [87]. The clinical-related advances in glycobiology are focused on the prognostic value of sialylated epitopes as markers of pathology. For example, it has been shown that serum sialic acid forecast both coronary heart disease and stroke fatality and reflects the progress of an atherosclerotic process [88,89,90]. The clinical observation and experimental models described an enhanced level of total serum sialic acid and sialoglycans on the human vein endothelial cells as a marker of pathology and significant risk factor of coronary artery disease (CAD) and angiopathy [91,92]. Moreover, the glycome analysis in asthma and COPD showed that the alterations in sialoglycans correspond to the progress of disease [93,94,95]. According to Guo, the lung pathology inducing factors, e.g., tobacco, can be considered as the crucial players in the promotion of SARS-CoV-2 infection and associated with the risk of severe COVID-19 [95]. Given the harmful effect of cigarette smoke (CS) on respiratory and circulatory parameters, heavy smokers are at higher risk of developing severe COVID-19 symptoms [96,97]. Beside the clinical consequences, CS induces multiple molecular changes in the lung cells. Our previous studies revealed that human alveolar epithelial A549 cells present enhanced α2-3- and α-2-8-, but not α2-6-sialylation pattern when exposed to CS [98]. Additionally, Leung et al. found that patients with chronic obstructive pulmonary disease (COPD) and individuals who are still smoking presents higher levels of ACE-2 in their airways [99]. Therefore, it is reasonable to hypothesize that dual-mechanism based on ACE2 and sialic acid might put them at an increased risk of developing severe COVID-19 infections.

### 2.5. Does Siglec-Sialic Acid Axis Participates in CoVs Infections?

The level of immune system activity is the major implication for the protection of body systems against pathogens. As multiple investigations indicate, the response in several individuals for exposure to the same pathogen is different ranging from asymptomatic course to “cytokine storm” and death. The most coronaviruses-related worse prognosis is due to the immune system becomes hypersensitive in its response, not direct virus-caused damage. It is well known that a difference in the way populations respond to infection is largely controlled by genetics that shapes immune profiles, however, the immune mechanisms engaged in CoVs infection, in particular SARS-CoV-2, are still not fully understood [100,101].

**Figure 2:**
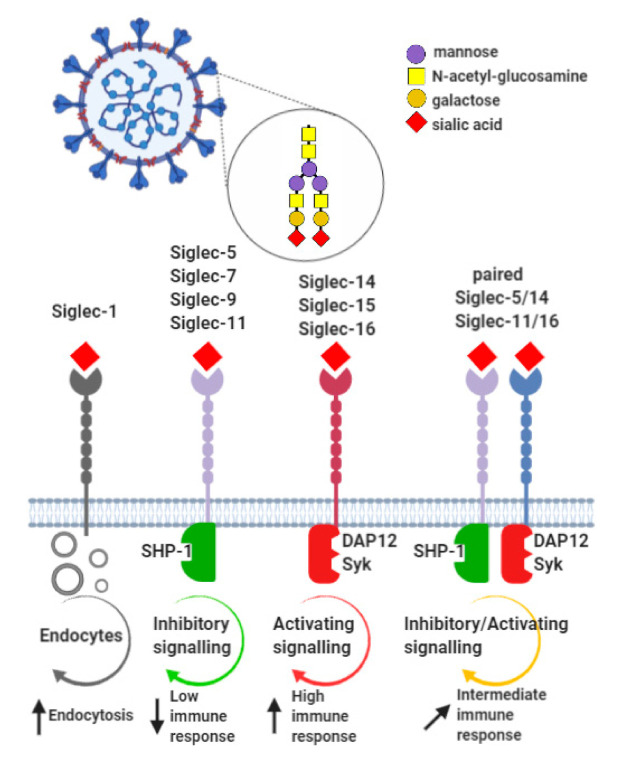
The possible effects of the CoVs targeting to Siglecs. The viral envelope glycans interact with sialic acid-binding lectins expressed in the host cell-dependent manner. Depending on the individual Siglec expression profile, the interactions with the viral sialic acid ligands exerts the immune response of different intensity. Abbreviations: DAP12-DNAX activation protein of 12 kDa; Syk-spleen tyrosine kinase; SHP-1-Src homology region 2 domain-containing phosphatase-1; SHP-2-Src homology region 2 domain-containing phosphatase-2.

In the context of participation of sialic acid in the mechanisms of CoVs entry, we discussed above scenario showed cellular sialoglycans as receptors for recognizing proteins expressed in the viral envelope. On the other hand, several pathogens express sialoglycan epitopes that act as a ligand recognized by cell membrane receptors [102] (Figure 2).

The virus-associated enzymes can exert modulatory effect on the cell membrane glycocalyx and unmask Siglecs by disruption of their *cis* ligands. It has been shown that unmasking of Siglec-1 (sialoadhesin, Sn) facilitate the CoV uptake into macrophages [103,104,105]. Another mechanism of potential importance includes interactions between sialic acids and Siglec receptors resulting in divergent immune system regulation. The role of CoV-originated sialoglycans in immune modulation was not extensively studied and the poor data have rather speculative value. Is it reasonable to consider the interplay between Siglecs and sialic acids as a controlling mechanism in CoVs invasion machinery? Varki and Angata hypothesize that expression of sialic acids by the envelope of CoV can affect Siglec receptors biology in the hosts and thereby regulate the reactivity of innate immune cells [102]. The inhibitory receptors, such as Siglec-5, -7 and -9, are implicated in molecular mimicry mechanism that allow several pathogens to avoid immunesurveillance as shown in the hepatitis B virus (HBV) infections [106,107]. In the context of Siglec-mediated immune regulation, the clinical differences in the CoVs infections can be interpreted by the variety of human phenotypes. Among the Siglec family members, the paired receptors express extremely similar amino acid sequence of extracellular domain and comparable tissue distribution resulting in the same ligand recognition capacity whereas the diverse intracellular signalling pathways trigger the opposite effects. The paired Siglec-5 and Siglec-14 proteins, gene fusion products, are widely expressed on myeloid-derived cells residing and infiltrating human airways, however their distribution in individuals is closely related to the frequencies of wild-type and SIGLEC-14 null alleles in various populations [108,109]. Therefore, the expression of activating Siglec-14 receptor predominates in the European population whereas inhibitory Siglec-5 is widely expressed in Asians. Clinically, the expression of Siglec-5 is closely linked to reduced bactericidal and virucidal abilities during infections with *Streptococcus*, *Neisseria*, *Pseudomonas*, *Campylobacter* and *HIV* [110]. Moreover, expression of Siglec-14 predispose to the potentiated inflammatory response in bacterial *(Haemophilus influenzae)* and viral infections (influenza virus), which are the cause of chronic respiratory diseases. It was confirmed by elevated expression of IL-1β in primary macrophages from SIGLEC14^+/+^, but not SIGLEC14^−/−^ individuals [111]. Interestingly, the expression and function of paired Siglec-5/14 receptors undergo multiple modification in response to several pathology promoting factors. In the experimental model of exposure to cigarette smoke (CS), an increase in the expression of paired Siglec-5/14 receptors in THP-1 cells was observed [98]. It was accompanied by simultaneous alterations in immune activity with an enhanced intracellular interleukin IL-1β and interleukin 10 (IL-10) expression, and reduced phagocytic capacity. Given the CS-induced functional alterations in the immune response, it is reasonable to speculate that sialic acid-Siglec axis can be an important mechanism of exacerbations in respiratory diseases and the immune system performance in viral infections in smokers.

Additionally, previous studies have revealed that conventional therapies may influence Siglec-mediated immune regulation. During the SARS epidemic in 2003, the early anti-inflammatory treatment of corticosteroids enhanced plasma viral titre leading to exacerbated disease [112]. The strong suppressive action of corticosteroids may explain these effect, however, Siglec-based mechanism can be also involved. The cytometric analysis of cell population isolated from patients with chronic obstructive pulmonary disease (COPD) has demonstrated that inhaled corticosteroids increase the expression of Siglec-5/14 in CD14+ cells [113]. Similarly, the enhancement of Siglec-9 in neutrophils was observed after management with dexamethasone (Dex) [114]. Therefore, the clinical assessment of Siglecs profiles can be considered before the therapeutic intervention with corticosteroids in individuals with respiratory comorbidities in CoVs infections.

Interestingly, the running clinical trials on the potential therapeutic management of COVID-19 showed that 4-aminoquinoline derivatives, chloroquine (CLQ) and hydroxychloroquine (CLQ-OH), prevent the virus from binding to ACE2 receptor [115]. Based on the in vitro studies, it is believed that chloroquine inhibits terminal glycosylation and thereby prevent SARS-CoV-2 from binding with gangliosides [116]. As structural and molecular modelling approaches have shown, this effect seems to be linked with direct interaction of chloroquine (CLQ) and hydroxychloroquine (CLQ-OH) with structural motif including amino acid residues 111–158 of the NTD that prevent of binding the sialylated GM1 with the high affinity [81]. From the molecular point of view, it may suggest the therapeutic potential of CLQ and CLQ-OH, however, the recent randomized trials of CLQ-OH as postexposure prophylaxis of COVID-19 did not fully confirm this action, therefore additional clinical and molecular observation are needed.

## 3. Conclusions and Perspectives

Although the biological importance of sialic acid is well described, its engagement in the pathogenesis of CoVs infections remains not fully understood. Recent findings in the field of virus attachment and entry to the host cells indicate that the recognizing capacity of defined sialoglycan epitopes becomes a distinctive feature among the coronaviruses family. In the most cases, the binding of sialic acid is a vulnerable part of dual mechanism of viral invasion, that could be targeted for the future development of therapeutics. On the other hand, the in vivo and in vitro studies on the role of sialic acid-Siglec axis can help the understanding of the immune-based pathogenesis of viral infection and its role in the divergent clinical course among the population, including the burden of asymptomatic individuals. The state of knowledge in this area is highly progressive, however, there are several questions in the field of glycobiology remain still unanswered: (i) Could sialic acid act as a host cellular marker to regulate the risk of CoVs attachment? (ii) Do several CoVs express sialic acids epitopes to regulate Siglec function? (iii) Is sialic acid-Siglecs interplay in CoVs infections relevant to cell function and activation? (iv) Are conventional drugs potential modulators of cellular sialome in the CoVs attachment process? (v) Do comorbidities and pathological factors associated with increased sialylation pattern predispose to SARS-CoV-2 infections and/or their severe course?

The prevention of viruses attachment and entry is the main challenge of modern virology. In the light of current SARS-CoV-2 pandemic, the novel immunotherapeutic approaches to COVID-19 should be developed in accordance to known and well established molecular mechanisms. Based on the role of sialoglycans in cell biology mentioned in this review, the targeting the patients’ sialome-modulating machinery may inhibit the viral invasion through multiple potential mechanisms of action including modifications of the host receptors, modulation of endocytosis, prevention of virus-host cell interaction, and immunomodulation.
